# Climate V. Care in the Treatment of Phthisis

**Published:** 1910-01-08

**Authors:** 


### The Hospital

A Journal of

The Medical Sciences and Hospital Administration.

New Series. No. 151, Vol. VI. [No. 1223, Vol. XLVII.] SATURDAY, JANUARY 8, 1910.

### CLIMATE v. CARE IN THE TREATMENT OF PHTHISIS.

In a recent communication to a contemporary Dr. S. Vere Pearson offers some very sensible suggestions upon what we may call the rational basis of treatment for consumption. The burden of his remarks is that at the present time medical men are inclined to make a fetish of climatic conditions, high altitudes, and so forth, to the neglect of the more intimate needs of the individual patient—needs determined by personal idiosyncrasy and by the precise characters of the lesion in any given case. This is not to say that climatic conditions are negligible. Dr. Pearson freely admits, and this from his own experience, that they are of considerable moment, but urges that they should be held distinctly subsidiary to the more important study of those individual symptoms upon a full and comprehensive appreciation of which success or failure so largely depends. This is no more than is to be expected when one considers dispassionately the nature of the malady. It is a recognised aphorism of treatment that inflamed structures need repose; yet an unguarded recommendation of some of the best known continental resorts for consumptives may lead a patient to a life which is far from reposeful. For the common age of incipient consumptives is an age of physical enthusiasm and vigour, and the danger of excess in the direction of physical exercise is one all too insufficiently realised. When a young adult, who has all his life enjoyed robust health, and has delighted in defying with impunity a large assortment of the usual rules of health, is suddenly asked to consider himself an invalid, and that on the score of symptoms which appear in his eyes extremely insignificant, it is no easy matter to bring home to him the urgent need for serious attention to the recovery of health. It will certainly not be managed by leading him to the belief that all he has to do is to winter abroad and enjoy himself, while climate works miracles in his damaged lung-tissues.

Of the symptoms supplying indications for prognosis and treatment, Dr. Pearson considers the temperature to be the most reliable, and insists that it should be habitually taken in the rectum, since relatively small variations in temperature may be most illuminating in the case of consumptive patients, and such slight variations may fail to be recorded if oral readings are made the rule. This appears to be particularly true of the temperature when taken after exercise. The temperature is to be taken four times during the day: at 7.45 a.m., 12 noon, 6.45 p.m., and 9.45 p.m., since important deductions can be made from the type of the temperature curve. It is, of course, vital that the readings should be correctly interpreted, and the interpretation offered by the author, who speaks with a large experience of sanatorium treatment, is as follows: Firstly, it must be borne in mind that minute variations may be very important in the treatment of consumption; and, secondly, that the temperature of the healthy body at rest is lower than is usually thought to be the case. The early morning reading is 98° F. or lower, and the evening reading 99.4° F. or lower, and any excess upon these figures is to be regarded as "fever" and as evidence of activity in the disease. Further, activity of the disease is an indication for as near an approach to complete rest for the lung as the nature of things will allow, and this can only be achieved by complete rest in bed. In cases when the temperature is persistently high, it is recommended that the patient be treated with as rigid care in the matter of active movement as though the disease were typhoid fever.

The type of the temperature curve has an important bearing upon prognosis. There are, according to the author, four principal types of phthisical temperature-curve. In the normal type the early morning reading is the lowest, and the highest between 3 and 8 p.m. In a second group the lowest reading is at 12 noon, instead of the first thing in the morning. In a third the highest reading is at 9.45 p.m. instead of 6.45 p.m. In a fourth the curve is quite erratic from day to day. Of these abnormal types the last is considered, from the prognostic point of view, to be the most unfavourable, while the first is more favourable than the second. From the standpoint of practice, therefore, patients whose temperature curves show a departure from the normal type require to be treated with more caution, especially in the matter of exercise, than those who exhibit a normal curve.

A point of considerable importance in interpreting the temperatures of female consumptives concerns the influence of menstruation. A slight elevation of temperature appears to be the rule in the case of women approaching the menstrual period. The practical bearing of the observation is that during these intervals a completer rest is necessary than may be called for at other times.

We have seen that Dr. Pearson places the greatest weight on the temperature as a guide to the propriety of making advances in the matter of exercise. We may abbreviate his advice on these points as follows: Exercise may be begun cautiously when the morning readings have fallen below 98.2° F., and the evening readings below 99.4° F.; but the golden rule is to proceed slowly, the increases in this direction being almost imperceptible from week to week. Exercise should never be increased on a rising temperature, but practice should not be guided by isolated features of the chart so much as by a general consideration of the types of curve afforded by the immediately preceding days. On rare occasions it may be well to depart from the rigid rule laid down, and institute mild exercise even in the absence of the temperature conditions formulated above, since in some stationary cases the mere appearance of an advance in progress has a stimulating mental effect.

We have given the chief points of Dr. Pearson's communication at some length because he deals in a practical way with problems which present themselves daily to every physician. He has certainly made out a good case against the common practice of discharging consumptives to some foreign health resort without sufficient consideration of the many circumstances which dictate success or failure in attempts to cure pulmonary consumption.

